# ASSOCIATION BETWEEN PHYSICAL ACTIVITY PRACTICE AND CLUSTERING OF HEALTH RISK BEHAVIORS IN ADOLESCENTS

**DOI:** 10.1590/1984-0462/2020/38/2018247

**Published:** 2020-02-14

**Authors:** Michael Pereira da Silva, Ana Beatriz Pacífico, Thiago Silva Piola, Edmar Roberto Fantinelli, Edina Maria de Camargo, Rosimeide Francisco Santos Legnani, Wagner de Campos

**Affiliations:** a Universidade Estadual do Centro-Oeste, Guarapuava, PR, Brazil.; b Universidade Federal do Paraná, Curitiba, PR, Brazil.; c Universidade Estadual de Ponta Grossa, Ponta Grossa, PR, Brazil.

**Keywords:** Physical activity, Risk behavior, Adolescents, Atividade física, Comportamento de risco, Adolescentes

## Abstract

**Objective:**

To verify the association between participation in physical activity (PA) and the clustering of health risk behaviors (HRB) in adolescents of both genders.

**Methods:**

A cross-sectional study involving 862 adolescents (11 to 17 years old) enrolled in 14 randomly selected public schools from Curitiba, Paraná, Brazil. Participation in PA, screen time, consumption of fruit, vegetables, cigarettes and alcoholic beverages were the criteria evaluated. Multinomial logistic regression tested the association between participation in PA and clustering of HRB, and results are expressed *Odds Ratio* (OR) with 95% confidence intervals (95%CI).

**Results:**

Adolescents with high participation in PA had lower odds of clustering 2-3 HRB (OR 0.38, 95%CI 0.21-0.68; p<0.01) and 4-5 HRB (OR 0.29; 95%IC 0.16-0.53; p<0.01). Boys with high participation in PA had lower chances of clustering 2-3 HRB (OR 0.31, 95%CI 0.13-0.75; p=0.01), and girls had lower odds of clustering 2-3 HRB (OR 0.41; 95%CI 0.17-0.99; p=0.04) and 4-5 HRB (OR 0.25; 95%CI 0.10-0.61; p<0.01).

**Conclusions:**

High participation in PA was inversely associated with the clustering of HRB in adolescents.

## RESUMO

**Objetivo:**

Verificar a associação entre a participação em atividade física (AF) e a agregação de comportamentos de risco à saúde (CRS) em adolescentes de ambos os sexos.

**Métodos:**

Estudo transversal com participação de 862 adolescentes (11 a 17 anos) matriculados em 14 escolas públicas, selecionadas aleatoriamente, da cidade de Curitiba, Paraná. Foram avaliados a participação em AF, o tempo em tela, o consumo de frutas, vegetais, cigarros e de bebidas alcoólicas por meio de questionários. Para a associação entre os níveis de participação em AF e a agregação dos CRS, foi utilizada a regressão logística multinominal com obtenção de razão de chances (RC) com intervalos de confiança de 95% (IC95%).

**Resultados:**

Os adolescentes com alta participação em AF apresentaram menores chances de agregação de 2-3 CRS (RC 0,38; IC95% 0,21-0,68; p<0,01) e 4-5 CRS (RC 0,29; IC95% 0,16-0,53; p<0,01). Meninos com alta participação em AF apresentaram menores chances de agregarem 2-3 CRS (RC 0,31; IC95% 0,13-0,75; p=0,01), e meninas, menores chances de agregação de 2-3 CRS (RC 0,41; IC95% 0,17-0,99; p=0,04) e 4-5 CRS (RC 0,25; IC95% 0,10-0,61; p<0,01).

**Conclusões:**

A alta participação em AF esteve inversamente associada com a agregação de CRS em adolescentes.

## INTRODUCTION

Behaviors such as insufficient physical activity (PA), low consumption of fruits and vegetables, high amounts of sedentary time, and the consumption of cigarettes and alcohol are important health risk factors and are increasingly common in adolescents. ^1^ ^,^ ^2^ ^,^ ^3^ ^,^ ^4^ Evidence shows that these behaviors tend to aggregate,^5^ ^,^ ^6^ potentially increasing the risk of morbidity and mortality.^7^ ^,^ ^8^

This aggregation seems to have a key period in adolescence, since in this phase of life, people go through a natural period of experimentation and they are faced with many opportunities to adopt health risk behaviors (HRB). Such behaviors tend to continue throughout early adulthood, which is the phase when health-related habits are solidified.^9^ Brazilian studies on specific risk behaviors with adolescents showed that about 50 to 60% of this population has at least two HRB. The main ones are sex, mother’s education level and economic condition.^6^ ^,^ ^10^

Insufficient PA is one of the risk behaviors that can negatively affect adolescents’ health. However, evidence suggests that regular PA, in addition to offering direct health benefits to adolescents, may also play an important role in adopting other related behaviors. In other words, more physically active adolescents tend to adopt healthier behaviors, such as adequate consumption of fruits and vegetables,^12^ ^,^ ^13^ less screen time^14^ and less drug use such as cigarettes and alcohol.^15^ ^,^ ^16^ ^,^ ^17^ These findings show how participation in PA favors other health-related behaviors, as it seems to be a behavior that stimulates a healthy lifestyle, bringing benefits to individuals’ health during adolescence with equally relevant adjustments into adulthood.^18^ On the other hand, the positive effects of PA on these behaviors are not consistent in the literature, as other studies suggest that more active adolescents are more susceptible to alcohol consumption^19^ ^,^ ^20^ and smoking cigarettes.^19^

As can be observed, several studies sought to identify the associations of PA with other behaviors in isolation. However, studies involving the aggregation of these HRB did not investigate the possible influence of habitual PA on these other aspects, thus limiting the verification of the importance of active lifestyle in the aggregation of HRB in adolescents. Therefore, this study aimed to verify the association between participation in PA and the aggregation of HRB in adolescents enrolled in the public-school system of Curitiba, Paraná.

## METHOD

This study used cross-sectional data regarding the first evaluation of the project “*Atividade Física e Comportamentos de Risco à Saúde em Adolescentes: Um Estudo de Coorte Prospectivo*” (Physical Activity and Health Risk Behaviors in Adolescents: A Prospective Cohort Study), initiated in 2015, with approval by the Research Ethics Committee of the Universidade Federal de Paraná (UFPR) (CAAE: 39206214.3.0000.0102). All participants presented an informed consent form that had been signed by their parents/guardians and an informed consent form that they themselves had signed.

The population was composed of adolescents (11 to 17 years old) of both sexes, enrolled in the public-school system of Curitiba, Paraná. The sample selection had two phases: the first one in clusters, respecting the representativeness of each educational region that belonged to the Curitiba Regional Education Center. From a random selection within each educational region, 14 schools were chosen. Each school provided 4-6 classes ranging from the sixth grade of elementary school to the first year of high school. At this stage, the classes were intentionally selected according to the logistics of the teaching staff of the educational institutions.

The present study obtained data from 930 participants, but only 862 presented valid data for this study, resulting in a loss of approximately 7.3%. The sample calculation was also performed for *a posteriori* hypothesis tests using the G*Power calculator. This sample number was sufficient to obtain odds ratios (OR) of equal to or less than 0.55 with a power of 80% and an alpha of 95%.

The Youth Activity Profile (YAP) questionnaire in its Brazilian version (available with authors) evaluated the levels of participation in PA. YAP is a seven-day reminder designed to measure PA in children and adolescents. It consists of ten multiple-choice PA items performed in different contexts. The associations of YAP with moderate to vigorous intensity physical activity (MVPA) measured objectively were positive and moderate (p <0.001).^21^ YAP was cross-culturally adapted to assess PA participation of Brazilian adolescents according to the recommendations of Van de Vijver and Hambleton,^22^ and its validity was tested using a sample of 137 Brazilian adolescents from age 11 to 17 years old. ^23^ The correlation between YAP scores and AFMV measured by accelerometry was moderate (rho: 0.44; p <0.001).

Questions regarding the use of active commuting to and from school, participation in PA before and after school hours, and on weeknights were asked according to weekly frequency (“no days” to “4-5 days”) of involvement in PA in these specific contexts. The questions regarding PA participation in Physical Education classes, during recess and breaks and during lunch break (full-time students only), asked about the level of participation in PA during these periods (e.g. “Almost nothing” to “Almost all the time”).

All questions were scored in a range from 1 to 5, with higher scores representing higher levels of participation in PA. YAP, in its original version, had the possibility of estimating time in MVPA based on participation scores, however the calibration process of this questionnaire in Brazilian adolescents is still ongoing. Therefore, in this study, the average score of the ten questions related to PA practice was used as a general indicator of MVPA. And to identify the level of participation in PA, tertiles were created indicating low, moderate and high participation in PA.

For HRB, screen time was assessed using the Sedentary Activity Questionnaire for Adolescents (*Questionário de Atividades Sedentárias para Adolescentes* - QASA),^24^ ^,^ ^5^ which provides information on time spent on different types of sedentary activity on the weekdays and weekends of a typical week. QASA has positive indicators of validity and reproducibility in Brazilian adolescents (ICC 0.88; 95% confidence interval [95% CI] 0.82-0.91; rho: 0.79; p <0.01).^25^ ^,^ ^26^ Teenagers who reported > 4 hours/day in front of a screen were considered in the category of high screen time.

The eating habits of adolescents assessed in the Youth Risk Behavior Survey (YRBS)^27^ were used for fruit and vegetable consumption. The present instrument was translated and validated (*Kappa*= 68.6%) for Brazilian students.^28^ Adolescents who reported a frequency of consuming fruits (fruits + natural juices) and vegetables <3 times a day were considered to be in the category of low consumption of these foods.

Cigarette and alcohol consumption among adolescents was assessed through questions from the Portuguese version of YRBS.^28^ Adolescents who reported consuming one or more cigarettes and five or more servings of alcohol at least once in the last 30 days prior to data collection were rated for the presence of risk behavior.

Based on the information reported by the adolescents regarding the evaluated HRB, they were divided into three groups according to the aggregation of these behaviors:

- 0-1 HRB.
- 2-3 HRB.
- 4-5 HRB.

The following covariates were analyzed: sexual maturation, nutritional status, economic classification, and education level of the head of household. Sexual maturation was assessed by the self-assessment method of pubic hair development with the aid of drawings.^29^ ^,^ ^30^ ^,^ ^31^ The procedure was guided by an evaluator of the same-sex as the responding participant. Nutritional status was assessed by calculating body mass index (BMI) obtained by the ratio of body mass (kg) to squared height (m). Nutritional status was identified by the BMI Z-score classifications for each sex and age proposed by the World Health Organization (WHO): underweight (<-2 SD); eutrophic (≥-2DP and <+1DP); overweight (≥ +1DP and <+2DP); and obese (≥ +2DP).^32^ Economic class was assessed by applying the Brazilian Economic Classification Criteria (*Critério de Classificação Econômica Brasil*) questionnaire proposed by the Brazilian Association of Research Companies (*Associação Brasileira de Empresas de Pesquisa* - ABEP).^33^ Adolescents were classified into high (class A), medium (classes B1 and B2) and low (classes C, D and E) socioeconomic levels (SEL). The question regarding the head of household’s level of education present in this questionnaire was also used in isolation as a covariate in the association between PA and HRB aggregation. For analysis purposes, this variable was divided into three categories:

- Low: “received no schooling” to “did not complete middle school”.
- Middle: completed elementary and high school but did not complete any higher education.
- High: completed higher education.

To describe the sample, a descriptive analysis was used by checking measures related to central tendency and dispersion (mean and standard deviation) for continuous variables and checking the distribution of absolute and relative frequency for categorical variables. Differences in HRB between the levels of participation in PA were tested using the chi-square test.

Binary logistic regression was used to verify the association between PA and HRB in isolation. To verify the association between PA and HRB aggregation, multinomial logistic regression was used. For all analysis models, the 95% CI adjusted for sex, age, nutritional status, economic classification and educational level of the head of household was obtained. Robust cluster weights and error control were used to avoid bias due to the complex sample selection pattern. All of the analyzes were performed using the STATA 13 MP software, and a p-value of <0.05 was adopted as the level of statistical significance.

## RESULTS

At the end of the study, 862 adolescents from 14 public schools in the city of Curitiba, Paraná, presented valid data, with an average age of 14.1±1.9 years (boys: 14.2±1.9 years; girls:13.9 ±1.9 years).

Table 1 presents results of the adolescents’ sociodemographic characteristics. Most of them were aged ≥14 years (55.2%), were eutrophic (61.2%), had medium SEL (56.8%) and a head of household with a low education level (52.5%). Comparisons by sex identified significant differences in nutritional status, in which boys had a higher prevalence of obesity when compared to girls (15.2 *versus* 10.9%; chi-square = 8.33; p = 0.01).

**Table 1:** Sociodemographic characteristics of adolescents from Curitiba, Paraná, Brazil (n = 862).

	Total (n = 862)	Boys (n=421)	Girls (n=441)	chi-square	p-value*
	n (%)	n (%)	n (%)		
Age group					
<14 years	386 (44.8)	183 (43.5)	203 (46.0)	0.57	0.45
≥14 years	476 (55.2)	238 (56.5)	238 (54.0)		
Sexual maturity					
Stage 1	17 (1.9)	7 (1.5)	10 (2.4)	28.26	<0.01
Stage 2	116 (13.5)	55 (13.0)	61 (13.9)		
Stage 3	263 (30.5)	96 (22.9)	167 (37.7)		
Stage 4	320 (37.1)	173 (41.1)	147 (33.2)		
Stage 5	146 (17.0)	90 (21.5)	56 (12.8)		
Nutritional state					
Eutrophic	528 (61.2)	265 (62.9)	263 (59.6)	8.33	0.01
Overweight	222 (25.8)	92 (21.9)	130 (29.5)		
Obese	112 (13.0)	64 (15.2)	48 (10.9)		
Socioeconomic level					
Low	178 (20.6)	86 (20.4)	92 (20.8)	1.43	0.49
Medium	490 (56.8)	233 (55.3)	257 (58.4)		
High	194 (22.6)	102 (24.3)	92 (20.8)		
Educational level of the head of household					
Low	453 (52.5)	213 (50.6)	240 (54.4)	4.86	0.08
Medium	243 (28.2)	133 (31.6)	110 (24.9)		
High	166 (19.3)	75 (17.8)	91 (20.7)		

*p <0.05 obtained by the chi-square test.

Table 2 presents the prevalence of participation levels in PA and HRB of the evaluated participants. Regarding PA, 35.5% of adolescents were classified has having low participation for the behavior. When it came to HRB, 48.6% of adolescents reported a high amount of time on screens; 95.6% presented low fruit consumption; 79.9% presented low consumption of vegetables; 15.3% consumed too much alcohol; and 6.3% smoked cigarettes in the 30 days prior to data collection. Regarding the aggregation of HRB, most participants presented at least two HRB (89.2%). Significant differences between the sexes were verified for participation in PA and time on screens. Boys were in the high PA group when compared to girls (43.5 *versus* 22.2%; chi-square = 54.59; p = 0.001). However, boys also reported more screen time compared to girls (57.2 *versus* 40.4%; Chi-square = 24.57; p = 0.003). No significant differences in sex were seen for HRB aggregation.

**Table 2:** Prevalence of participation in physical activity and health risk behaviors of adolescents in the city of Curitiba, Paraná, Brazil (n = 862).

	Total (n=862)	Boys **(n=421)**	Girls (n=441)	chi-square	p-value*
	n (%)	n (%)	n (%)		
Participation in PA					
Low	306 (35.5)	106 (25.2)	200 (45.4)	54.59	<0.01
Medium	275 (31.9)	132 (31.3)	143 (32.4)		
High	281 (32.6)	183 (43.5)	98 (22.2)		
Screen time					
<4 hours	443 (51.4)	180 (42.8)	263 (59.6)	24.57	<0.01
≥4 hours	419 (48.6)	241 (57.2)	178 (40.4)		
Fruit consumption					
<3 times/day	824 (95.6)	399 (94.8)	425 (96.4)	1.30	0.25
≥3 times/day	38 (4.4)	22 (5.2)	16 (3.6)		
Vegetable consumption					
<3 times/day	173 (20.1)	73 (17.3)	100 (22.7)	3.82	0.05
≥3 times/day	689 (79.9)	348 (82.7)	341 (77.3)		
Excessive alcohol consumption					
No	730 (84.7)	365 (86.7)	365 (82.8)	2.56	0.11
Yes	132 (15.3)	56 (13.3)	76 (17.2)		
Cigarette consumption					
No	808 (93.7)	398 (94.5)	410 (93.0)	0.89	0.34
Yes	54 (6.3)	23 (5.5)	31 (7.0)		
HRB aggregation					
0-1	93 (10.8)	37 (8.8)	56 (12.7)	3.55	0.17
2-3	700 (81.2)	351 (83.4)	349 (79.1)		
4-5	69 (8.0)	33 (7.8)	36 (8.1)		

PA: physical activity; HRB: health risk behaviors; *p <0.05 obtained by the chi-square test.

Results regarding the association between participation in PA and HRB alone can be seen in Table 3. In the general sample, it was found that participants in the group with high participation in PA had lower chances of low fruit consumption (OR 0.48; 95% CI 0.25-0.95; p = 0.03) and vegetables (OR 0.40; 95% CI 0.23-0.68; p <0.01) when compared to the group with low participation in PA. On the other hand, participation in PA was positively associated with excessive alcohol consumption. Adolescents with medium (OR 1.81; 95% CI 1.03- 3.17; p = 0.04) and high (OR 1.74; 95% CI 1.11- 2.74; p = 0.01) participation in PA were more likely to report this risky behavior as well.

**Table 3:** Binary logistic regression of the association between participation in physical activity and health risk behavior in adolescents from Curitiba, Paraná, Brazil (n = 862).

Participation in PA	Screen time **(≥4 horas)**		Low consumption of fruits		Low consumption of vegetables		Excessive alcohol consumption		Cigarette consumption	
	OR (95% CI)	p-value	OR (95% CI)	p-value	OR (95% CI)	p-value	OR (95% CI)	p-value	OR (95% CI)	p-value
Boys & Girls* (n = 862)										
Low	1		1		1		1		1	
Medium	0.76 (0.56-1.01)	0.06	0.56 (0.27-1.15)	0.12	0.78 (0.53-1.18)	0.20	1.81 (1.03-3.17)	0.04	0.73 (0.9-2.78)	0.64
High	0.64 (0.38-1.09)	0.10	0.48 (0.24-0.95)	0.03	0.40 (0.23-0.68)	<0.01	1.74 (1.11-2.74)	0.01	0.48 (0.18-1.26)	0.14
Boys** (n=421)										
Low	1		1		1		1		1	
Medium	0.53 (0.31-0.91)	0.02	0.25 (0.05-1.22)	0.08	0.73 (0.38-1.40)	0.35	3.85 (1.24-11.95)	0.02	1.64 (0.37-7.26)	0.66
High	0.43 (0.21-0.90)	0.02	0.29 (0.08-1.02)	0.05	0.58 (0.26-1.24)	0.16	3.75 (1.49-9.44)	<0.01	0.56 (0.14-2.19)	0.40
Girls** (n=441)										
Low	1		1		1		1		1	
Medium	0.92 (0.61-1.37)	0.68	0.78 (0.15-4.16)	0.77	0.84 (0.55-1.28)	0.42	1.24 (0.50-3.05)	0.63	0.47 (0.10-2.29)	0.35
High	0.85 (0.46-1.57)	0.61	0.69 (0.14-3.41)	0.65	0.32 (0.18-0.54)	<0.01	1.19 (0.63-2.25)	0.58	0.68 (0.14-3.21)	0.63

PA: physical activity; OR: odds ratio; 95% CI: 95% confidence interval; *adjusted for sex, age group, sexual maturation, nutritional status, socioeconomic status and education level of the head of household; ** adjusted for age group, nutritional status, sexual maturation, socioeconomic status and education level of the head of household.

For analyzes stratified by sex, inverse associations between participation in PA and HRB alone were verified for screen time in boys and for low vegetable intake in girls. Boys with medium (OR 0.53; 95% CI 0.31-0.91; p = 0.02) and boys with high (OR 0.43; 95% CI 0.21-0.90; p = 0.02) levels of participation in PA were less likely to also show screen time ≥4 hours per day. In relation to girls, those with high participation in PA were less likely to indicate low vegetable consumption (OR 0.32, 95% CI 0.18-0.54; p <0.01).

Regarding the associations between participation in PA and excessive alcohol consumption, positive associations were identified only for males. Adolescents with medium (OR 3.85; 95% CI 1.24- 3.85; p = 0.02) and high (OR 3.75; 95% CI 1.49- 9.44; p<0.01) participation in PA were more likely to report this risky behavior as well.

The association between PA participation and HRB aggregation can be seen in Table 4. OR and 95% CI were obtained for both the total sample and for each sex. For the general sample, it was found that adolescents who were in the group with high levels of participation in PA were less likely to have 2-3 HRB (OR 0.38; 95% CI 0.21-0.68; p <0.01) and 4-5 HRB (OR 0.29; 95%CI 0.16-0.53; p <0.01), when compared to the group with low levels of participation in PA. By stratifying the analysis for each sex, it was observed that boys with high levels of participation in PA had lower chances of aggregating 2-3 HRB (OR 0.31; 95% CI 0.13-0.75; p = 0.01), when compared to boys with low levels of participation in PA. In turn, girls showed inverse associations between high levels of participation in PA and HRB aggregation for both 2-3 HRB (OR 0.41; 95% CI 0.17-0.99; p = 0.04) and for 4-5 HRB (OR 0.25; 95% CI 0.10-0.61; p <0.01), when compared to girls with low levels of participation in PA.

**Table 4:** Multinomial logistic regression of the association between participation in physical activity and aggregation of health risk behavior in adolescents from Curitiba, Paraná, Brazil (n = 862).

Participation in PA	Boys & Girls*				Boys**				Girls**			
	2-3 HRB		4-5 HRB		2-3 HRB		4-5 HRB		2-3 HRB		4-5 HRB	
	OR (95% CI)	p-value	OR (95% CI)	p-value	OR (95% CI)	p-value	OR (95% CI)	p-value	OR (95% CI)	p-value	OR (95% CI)	p-value
Low	1		1		1		1		1		1	
Medium	0.69 (0.38-1.25)	0.22	0.70 (0.29-1.69)	0.43	0.40 (0.09-1.76)	0.23	0.87 (0.19-3.95)	0.86	0.91 (0.50-1.66)	0.76	0.56 (0.14-2.29)	0.42
High	0.38 (0.21-0.68)	<0.01	0.29 (0.16-0.53)	<0.01	0.31 (0.13-0.75)	0.01	0.39 (0.14-1.06)	0.07	0.41 (0.17-0.99)	0.04	0.25 (0.10-0.61)	<0.01

HRB: health risk behaviors; PA: physical activity; OR: odds ratio; 95% CI: 95% confidence interval; *adjusted for sex, age group, sexual maturation, nutritional status, socioeconomic status and education level of the head of household; ** adjusted for age group, nutritional status, sexual maturation, socioeconomic status and education level of the head of household.

## DISCUSSION

The present study verified the association between participation in PA and the aggregation of HRB in 862 students enrolled in 14 state schools in the city of Curitiba, Paraná. The adoption of HRB in adolescence is an important public health problem due to its association with morbidity and mortality.^7^ ^,^ ^8^

In the present sample, there was a higher occurrence of obesity in boys compared to girls. These results differ from those presented by Guedes et al.^34^ in a cross-sectional study conducted with 4,319 children and adolescents from seven to 18 years old. In this study, the authors found that, regardless of age, girls were more likely to be both overweight and obese. However, this study was published in 2006, and used a different criterion than the one used in the present study for the classification of being overweight and obese, factors that may explain these possible divergences. On the other hand, corroborating the findings of the present study, more recent data from the Brazilian Adolescent Cardiovascular Risk Study (*Estudo de Riscos Cardiovasculares em Adolescentes brasileiros* - ERICA) identified a higher prevalence of obesity in boys (9.2%; 95% CI 8.4-9.9) compared to girls (7.6%; 95% CI 7.1-8.3).^35^

Regarding participation in PA and sedentary behavior between the sexes, the results of the present study indicate that boys were more engaged in both types of behavior, that is, they were more active and also spent more time in front of screens than girls. The literature corroborates these differences between boys and girls in adolescence and also indicates that the coexistence of these two behaviors, as seen in boys, is likely to occur, since the association of PA with this type of sedentary behavior is low. ^36^

The high prevalence of high amounts of screen time, low consumption of fruits and vegetables, excessive alcohol consumption, and cigarette smoking was worrisome because they indicated a less than healthy behavioral pattern in the adolescents studied. Such high rates increase the likelihood of these adolescents presenting multiple HRB, which play an important role in risk factors for obesity and cardiometabolic diseases.^37^ ^,^ ^38^

The presence of multiple HRB enhances health problems, and a high prevalence of the aggregation of these behaviors has been documented in Brazilian adolescents. Brito et al.,^10^ for example, identified that 58.5% of adolescents had at least two HRB. Mazzardo et al.,^6^ in a sample of adolescents from Curitiba, Paraná, identified that this pattern of HRB aggregation occurred in 50.7% of adolescents. Data from the present study show a more worrying scenario, since most participants (89.2%) had at least two HRB. More specifically, 81.2% of them had 2-3 HRB, and 8% had 4-5 HRB.

PA is an important modulating health behavior, regardless of age group. Some studies have shown that the level of PA in adolescents seems to be associated with the adoption of healthier behaviors, such as lower alcohol consumption and cigarette smoking, better nutritional habits and shorter amounts of sedentary time.^12^ ^,^ ^13^ ^,^ ^14^ ^,^ ^15^ ^,^ ^16^ ^,^ ^17^

In the present study, the analyzes of the association between participation in PA and the HRB alone identified that adolescents with high levels of participation in this behavior had healthier eating behaviors with regard to fruit and vegetable consumption. These findings corroborate studies that indicate participation in PA as an important predictor of adequate consumption of fruits and vegetables.^12^ ^,^ ^39^ In analyzing these associations for each sex, it was found that they remained significant only for girls’ consumption of vegetables. These findings indicate that more active adolescents tend to adopt better eating habits, and females are more prone to do this.

In contrast to what was presented for fruit and vegetable consumption, adolescents that were more engaged in PA were more likely to also present excessive consumption of alcoholic beverages. This association was more present in boys than in girls. The literature regarding the association of PA with alcohol consumption by adolescents presents controversial results.^15^ ^,^ ^16^ ^,^ ^17^ ^,^ ^19^ ^,^ ^20^ The mechanism by which the practice of PA can influence alcohol consumption is unclear. However, factors related to the greater social interaction resulting from this practice,^40^ as well exercising in places that have alcohol more readily available, may explain these results.

When dealing with HRB aggregation, the results of the present investigation show that high participation in PA is inversely associated with the presence of multiple HRB in the studied adolescents. The most active adolescents were 62 and 71% less likely to be in the 2-3 HRB and 4-5 HRB group, respectively, when compared to the least active. These results indicate the importance of adolescents in participating in PA in order to have a healthier lifestyle. Active people may be more aware of healthier habits, which is an important factor for having less multiple risk behaviors in this group.^41^

Additionally, the analysis performed for each sex showed more consistent results for girls. Female adolescents with high levels of participation in PA were 59% less likely to have 2-3 HRB and 75% reported at least 4-5 aggregated HRB. In boys, associations were seen only with the aggregation of 2-3 HRB, with 69% less chance for those with high participation in PA. HRB aggregation data from the National Longitudinal Study of Adolescent to Adult Health identified a high prevalence of HRB aggregation, especially in boys.^9^ The fact that the associations between PA and HRB aggregation are more consistent in adolescents can be explained by the more active girls’ awareness of healthier habits.

Data from the present study provide important information on how health-related behaviors can contribute to the adoption of a certain unhealthy habits and favor the adoption of other behaviors. On the other hand, the aggregation of HRB shows that behaviors that are considered to be healthy, such as PA, seem to favor lower engagement in multiple unhealthy behaviors. Thus, it could be a possible instrument of intervention that aims to prevent or reduce multiple HRB.

Some limitations of the study, such as the use of questionnaires to assess PA and sedentary behavior, may favor the overestimation of the results. However, in an attempt to minimize this bias, researchers were trained prior to data collection to assist adolescents in answering the questionnaires to the best of their ability. The assessment of fruit and vegetable consumption, alcohol consumption and cigarette smoking were restricted to seven and 30 days prior to data collection, respectively.

The cutoff point used for screen time classifications may limit the comparison of results with other studies. However, the commonly used two-hour cutoff point suggested by the American Academy of Pediatrics ^42^ in 2001 only covers TV time, which does not fully encompass all of the sedentary behaviors assessed by the instrument used. In the present study, screen time is presented as the combination of time watching TV, playing video games and using the computer, which could inflate the risk category of this component. Therefore, we decided to use ≥4 hours as a cutoff point because it approached the 50th percentile of the data distribution. Another important point to highlight as a possible limitation was the separate analysis of fruits and vegetables. Some studies combine these behaviors,^12^ but the analysis and presentation of them separately are supported by the Centers for Disease Control and Prevention.^27^ As a strong point, we highlight the random selection of 14 schools within nine regional schools, favoring the inclusion of adolescents from different social classes and residents in different regions of the city.

It can be concluded, in general, that adolescents with high levels of participation in PA were less likely to have HRB aggregation. The stratified analysis showed that more active boys were less likely to have 2-3 HRB aggregation. For girls, the association between high participation in PA with HRB aggregation occurred for both 2-3 HRB and 4-5 HRB.

The promotion of an active lifestyle is the target of global public policies aimed at improving the health conditions of populations of various age groups. The findings of the present study reinforce this need to promote and facilitate greater engagement of adolescents in doing PA in order to stimulate the adoption of other healthy behaviors.
